# Genetic Variations of Three Kazakhstan Strains of the SARS-CoV-2 Virus

**DOI:** 10.3390/v17030415

**Published:** 2025-03-14

**Authors:** Bekbolat Usserbayev, Kulyaisan T. Sultankulova, Yerbol Burashev, Aibarys Melisbek, Meirzhan Shirinbekov, Balzhan S. Myrzakhmetova, Asankadir Zhunushov, Izat Smekenov, Aslan Kerimbaev, Sergazy Nurabaev, Olga Chervyakova, Nurlan Kozhabergenov, Lesbek B. Kutumbetov

**Affiliations:** 1 Research Institute for Biological Safety Problems, National Holding QazBioPharm, LLP, Guardeyskiy uts 080409, Kazakhstan; k.sultankulova@biosafety.kz (K.T.S.); y.burashev@biosafety.kz (Y.B.); a.melisbek@biosafety.kz (A.M.); m.shirinbekov@biosafety.kz (M.S.); b.myrzakhmetova@biosafety.kz (B.S.M.); a.kerimbayev@biosafety.kz (A.K.); s.nurabayev@biosafety.kz (S.N.); o.chervyakova@biosafety.kz (O.C.); l.kutumbetov@biosafety.kz (L.B.K.); 2 Institute of Biotechnology, National Academy of Science of Kyrgyzstan, Bishkek 720071, Kyrgyzstan; 3 Scientific Research Institute of Biology and Biotechnology Problems, al-Farabi Kazakh National University, Almaty 050040, Kazakhstan; smekenovizat@gmail.com

**Keywords:** genome sequencing, Sanger method, mutation, phylogenetic analysis, COVID-19

## Abstract

Prompt determination of the etiological agent is important in an outbreak of pathogens with pandemic potential, particularly for dangerous infectious diseases. Molecular genetic methods allow for arriving at an accurate diagnosis, employing timely preventive measures, and controlling the spread of the disease-causing agent. In this study, whole-genome sequencing of three SARS-CoV-2 strains was performed using the Sanger method, which provides high accuracy in determining nucleotide sequences and avoids errors associated with multiple DNA amplification. Complete nucleotide sequences of samples, KAZ/Britain/2021, KAZ/B1.1/2021, and KAZ/Delta020/2021 were obtained, with sizes of 29.751 bp, 29.815 bp, and 29.840 bp, respectively. According to the COVID-19 Genome Annotator, 127 mutations were detected in the studied samples compared to the reference strain. The strain KAZ/Britain/2021 contained 3 deletions, 7 synonymous mutations, and 27 non-synonymous mutations, the second strain KAZ/B1.1/2021 contained 1 deletion, 5 synonymous mutations, and 31 non-synonymous mutations, and the third strain KAZ/Delta020/2021 contained 1 deletion, 5 synonymous mutations, and 37 non-synonymous mutations, respectively. The variations C241T, F106F, P314L, and D614G found in the 5′ UTR, *ORF1ab*, and *S* regions were common to all three studied samples, respectively. According to PROVEAN data, the loss-of-function mutations identified in strains KAZ/Britain/2021, KAZ/B1.1/2021, and KAZ/Delta020/2021 include 5 mutations (P218L, T716I, W149L, R52I, and Y73C), 2 mutations (S813I and Q992H), and 8 mutations (P77L, L452R, I82T, P45L, V82A, F120L, F120L, and R203M), respectively. Phylogenetic analysis showed that the strains studied (KAZ/Britain/2021, KAZ/B1.1/2021, and KAZ/Delta020/2021) belong to different SARS-CoV-2 lineages, which are closely related to samples from Germany (OU141323.1 and OU365922.1), Mexico (OK432605.1), and again Germany (OV375251.1 and OU375174.1), respectively. The nucleotide sequences of the studied SARS-CoV-2 virus strains were registered in the Genbank database with the accession numbers: ON692539.1, OP684305, and OQ561548.1.

## 1. Introduction

Severe acute respiratory syndrome coronavirus 2 (SARS-CoV-2), the causative agent of coronavirus disease 2019 (COVID-19), was first reported in December 2019 in Wuhan, Hubei Province, China [1]. According to WHO COVID-19 data, as of 23 February 2025, a total of 777,519,152 confirmed cases have been reported, of which 7,090,776 have resulted in death [2]. The first cases of COVID-19 in the Republic of Kazakhstan were reported on 13 March 2020 [3,4].

The SARS-CoV-2 virion is spherical or ellipsoidal, with an average diameter ranging from 60 to 140 nanometers [5]. The SARS-CoV-2 virus genome consists of ~29.9 kb and is organized in the following order from 5′ to 3′: open reading frame *(ORF) 1ab (replicase)*, structural spike glycoprotein *(S)*, *ORF3a* protein, structural envelope protein *(E)*, structural membrane glycoprotein *(M)*, *ORF6* protein, *ORF7a* protein, *ORF7b* protein, *ORF8* protein, structural nucleocapsid phosphoprotein *(N)*, and *ORF10* protein [6]

Over time, all viruses, including SARS-CoV-2, undergo molecular genetic changes. Most of these changes have little effect on the properties of the virus. However, some mutations can affect various aspects, such as its infectivity, transmissibility, the effectiveness of treatment and vaccines, as well as virulence [7]. In addition, since its emergence in 2019, the SARS-CoV-2 virus has undergone continuous changes, which contributed to the emergence of multiple lineages and variants (Alpha (B.1.1.7), Beta (B.1.351), Gamma (P.1), Delta (B.1.617.2), and Omicron (B.1.1.529)) [8,9], having differences in transmission characteristics, ability to cause severe disease, and ability to evade immune response [10].

The expansion of the complete genomic sequences of the SARS-CoV-2 virus in the information databases (GISAID and GenBank NCBI) was made possible by rapid genome sequencing using Sanger or NGS methods [11]. These SARS-CoV-2 genomes in the context of the COVID-19 pandemic can provide invaluable information on the evolution of the virus and allow tracking of the geographic distribution of individual mutations, as well as monitoring of the spread of the virus in the human population [12]. In addition, the evolution of the virus is facilitated by the adaptation of the virus in different conditions and results from a balance between its genetic information and genome variability [13]. Studying the evolution and genetic changes in the genomes of various variants of SARS-CoV-2 is extremely important in developing clinical and political strategies within geographical regions [14] as well as for the creation of diagnostic tests and vaccines against this virus [15].

The aim of this work is to sequence the complete genome of three isolates of the SARS-CoV-2 virus, determine their genetic variations, and identify various types of mutations present in the different strains.

## 2. Materials and Methods

### 2.1. Sample Collection

Three strains of the SARS-CoV-2 virus were received for molecular genetic studies at the Research Institute for Biological Safety Problems in 2022 from the Scientific and Practical Center for Sanitary and Epidemiological Expertise and Monitoring branch of the National Center for Public Health, a republican state enterprise on the right of economic use of the Ministry of Health of the Republic of Kazakhstan.

### 2.2. RNA Extraction

Total RNA was extracted from virus-containing fluid using the QIAamp Viral RNA Mini Kit (Qiagen, Hilden, Germany) according to the manufacturer’s instructions. RNA concentrations were estimated using Qubit RNA HS assay kits (Life Technologies, Carlsbad, CA, USA) on a Qubit 2.0 fluorometer (Life Technologies, Carlsbad, CA, USA) according to the manufacturer’s protocol.

### 2.3. cDNA Synthesis

Reverse transcription (RT) was performed using the SuperScript VILO cDNA Synthesis Kit (Invitrogen, Thermo Fisher Scientific, Carlsbad, CA, USA) in a Mastercycler X50 s thermal cycler at the following conditions: 25 °C for 10 min; 42 °C for 60 min; 85 °C for 5 min. The reaction composition and temperature–time conditions were followed according to the manufacturer’s instructions.

### 2.4. Primer Design and Synthesis

Specific overlapping primers for amplification and sequencing of all SARS-CoV-2 virus genes were manually searched and designed on the NCBI website using the GenBank database. The nucleotide sequence of the sequencing primers was designed based on the SARS-CoV-2 isolate Wuhan-Hu-1 reference strain (NC_045512.2) [16]. The specificity of the primers was checked using NCBI Primer-BLAST [17]. The primers were designed so that each pair overlapped each other, and their sequences were conserved in all SARS-CoV-2 virus variants. As a result, 65 pairs of sequencing primers were selected to amplify the complete genome of SARS-CoV-2 virus variants with an overlap of about 100 nucleotide base pairs (bp). The estimated length of the amplicons ranged from 600 to 772 bp [18]. Oligonucleotides were synthesized on an automatic DNA/RNA Synthesizer H-16 oligonucleotide synthesizer (K&A Labs GmbH, Schaafheim, Germany) using the phosphoramidite method performed according to the manufacturer’s protocol. The synthesized primers were eluted from the columns with a concentrated ammonia solution. The primers were then dried on a rotary evaporator and purified by alcohol precipitate.

### 2.5. Polymerase Chain Reaction (PCR) Setup

Amplification was performed on a Mastercycler X50 s thermal cycler using the Platinum SuperFi PCR Master Mix kit (Invitrogen, Thermo Fisher Scientific, Vilnius, Lithuania) according to the manufacturer’s instructions. PCR was performed in a total volume of 25 µL, composed of: 12.5 µL of 2X Platinum SuperFi PCR Master Mix, 1.25 µL of each of 10 µM forward and reverse primers, 3 µL of cDNA template, 5 µL of 5X SuperFi GC Enhancer, and PCR-grade water to bring the volume to 25 µL. PCR products were amplified using the following conditions: initial denaturation 95 °C—0.5 min; with subsequent 35 amplification cycles with denaturation at 95 °C for 0.1 min, annealing at 57 °C for 0.5 min, elongation at 72 °C for 0.5 min; final elongation at 72 °C for 5 min.

Horizontal gel electrophoresis was performed in 1.5% agarose gel (TopVision Agarose, Thermo Fisher Scientific Baltics, UAB, Vilnius, Lithuania) stained with ethidium bromide in Tris-acetate buffer at a voltage of 100 volts/cm of gel length for 30 min. The gel was subsequently viewed using a MiniBIS Pro transilluminator (DNR Bio Imaging Systems, Ltd., Jerusalem, Israel). Visualization and documentation of gel electrophoresis results were performed using the GelCapture program (DNR Bio-Imaging Systems Ltd., Ha-Satat St, Modi’in-Maccabim-Re’ut, Israel). A 100 bp DNA Ladder (New England Biolabs, Ipswich, MA, USA) was used as a molecular mass marker. The PCR product was purified using the GeneJET PCR Purification Kit (Thermo Fisher Scientific, Carlsbad, CA, USA) according to the manufacturer’s instructions.

### 2.6. Determination of Nucleotide Sequences

Sequencing of the whole SARS-CoV-2 virus genome after purification of the PCR product was carried out using termination dideoxynucleotides (Sanger method) with the AB BigDye Terminator v3.1 Cycle Sequencing Kit (Applied Biosystems, Inc., Austin, TX, USA) and specific overlapping primers designed from different viral genes used in the amplification step. The products were purified using the BigDye Xterminator kit (Applied Biosystems, Foster City, CA, USA) and sequenced using a 3130 XL Genetic Analyzer (HITACHI, Tokyo, Japan). After sequencing, the obtained nucleotide sequence data were processed using the Sequencher v.5.4 program (Gene Codes Corporation, Ann Arbor, MI, USA).

### 2.7. Lineage Determination and Mutation Identification of the Studied Isolates

The SARS-CoV-2 virus strain lineage determination was performed using the Pangolin COVID-19 database [19]. The alignment of the SARS-CoV-2 virus nucleotide sequences with the reference strain and the identification of mutations were performed using the COVID-19 Genome Annotator Tool and Annotator [20].

### 2.8. Analysis of Non-Synonymous Mutation Function

PROVEAN v1.1 software was used to determine whether the selected mutations independently resulted in potential loss of function or a neutral effect. Mutation scores above the default threshold of −2.5 imply a neutral effect, while scores below this threshold indicate a deleterious effect [21].

### 2.9. Phylogenetic Analysis of Nucleotide Sequences

Evolutionary analysis was performed in MEGA 11 [22]. A phylogenetic tree including three samples, a reference genome, and genomes of different SARS-CoV-2 lineages was constructed using the Neighbor-Joining method [23]. The percentage of replicates in which related taxa were grouped together in a bootstrap test (1000 replicates) was shown next to the branches [24]. The tree was drawn to scale, with branch lengths (next to the branches) in the same units as the evolutionary distances used to construct the phylogenetic tree. Evolutionary distance was calculated using the maximum composite likelihood method [25] and expressed in units of base substitutions per site. To construct the phylogenetic tree, sequences were first aligned in the GenBank database and viral nucleotide sequences that were similar to the sequence of the strain under study were selected. The most suitable substitution model was selected for tree construction. A preliminary tree was constructed using the appropriate model. Then, the tree was pruned, typical strains were selected by year and territory, and the lineage of the strain under study was determined. When constructing a new phylogenetic tree, strains of another lineage were selected.

## 3. Results

### 3.1. PCR Amplification of SARS-CoV-2 Virus Strains

**Figure 1:**
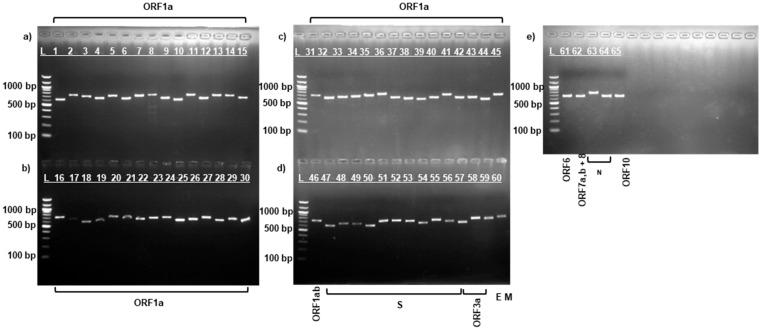
Electrophoresis of amplified fragments of all genes of the SARS-CoV-2/human/KAZ/Britain/2021 strain. Upper and lower gel (**a**–**d**): lanes 1 to 46—*ORF1ab gene*; lower gel (**d**): Lanes 47 to 56—*S gene*; Lanes 57 to 58—*ORF3a gene*; Lane 59—*E gene*; Lane 60—*M gene*; upper gel (**e**): Lane 61—*ORF6 gene*; Lane 62—*ORF7a*, *ORF7b*, and *ORF8*; Lanes 63 to 64—*N gene*; Lane 65—*ORF10 gene*; Lane M—100 bp DNA marker.

**Figure 2:**
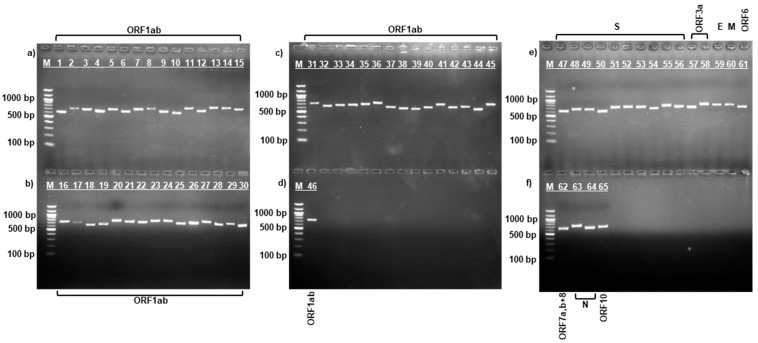
Electrophoresis of amplified fragments of the entire gene of the SARS-CoV-2/human/KAZ/B1.1/2021 strain. Upper and lower gel (**a**–**d**): Lanes 1 to 46—*ORF1ab gene*; upper gel (**e**): Lanes 47 to 56—*S gene*; Lanes 57 to 58—*ORF3a gene*; Lane 59—*E gene*; Lane 60—*M gene*; Lane 61—*ORF6 gene*; lower gel (**f**): Lane 62—*ORF7a*, *ORF7b*, and *ORF8 genes*; Lanes 63 to 64—*N gene*; Lane 65—*ORF10 gene*; Lane M—100 bp DNA marker.

**Figure 3:**
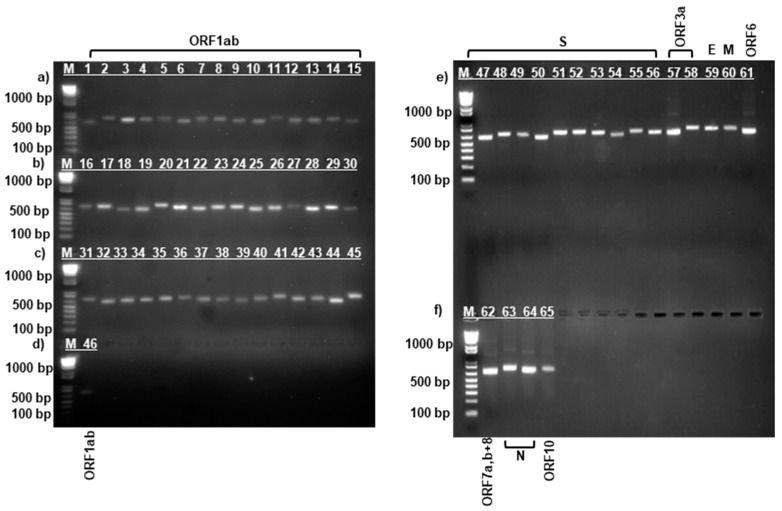
Electrophoresis of amplified fragments of the entire gene of the SARS-CoV-2/human/KAZ/Delta020/2021 strain. Upper and lower gel (**a**–**d**): lanes 1 to 46—*ORF1ab gene*; upper gel (**e**): lanes 47 to 56—*S gene*; lanes 57 to 58—*ORF3a gene*; lane 59—*E gene*; lane 60—*M gene*; lane 61—*ORF6 gene*; lower gel (**f**): lane 62—*ORF7a*, *ORF7b* and *ORF8 genes*; lanes 63 to 64—*N gene*; lane 65—*ORF10 gene*; lane M—100 bp DNA marker.

After RNA extraction and cDNA synthesis, amplification was performed using specific sequencing primers [18] for the complete SARS-CoV-2 virus genome by PCR according to the manufacturer’s protocol described above. Figure 1, Figure 2 and Figure 3 show the results of the electropherogram using the developed 65 pairs of sequencing primers.

As can be seen in Figure 1, Figure 2 and Figure 3, fragments of the complete genome of the SARS-CoV-2 virus samples were obtained using the developed sequencing primers [18]. Electrophoretic analysis yielded products with a molecular weight ranging between 612–732 bp. The length of the obtained amplicons corresponds to the length of the synthesized sequencing primers.

### 3.2. Characteristics of the Genomes of the Studied SARS-CoV-2 Virus Strains

The size of the genomes of the studied samples SARS-CoV-2/human/KAZ/Britain/2021 (KAZ/Britain/2021), SARS-CoV-2/human/KAZ/B1.1/2021 (KAZ/B1.1/2021), and SARS-CoV-2/human/KAZ/Delta-020/2021 (KAZ/Delta020/2021) were 29.751 bp, 29.815 bp, and 29.840 bp, respectively, and the GC contents were 38%, 37.95%, and 38%, respectively [4,26,27].

The nucleotide sequences of Kazakhstan SARS-CoV-2 virus strains were analyzed using the Pangolin COVID-19 database [19]. According to Pangolin COVID-19 data, the studied strains KAZ/Britain/2021, KAZ/B1.1/2021, and KAZ/Delta020/2021 belong to the B.1.1.7, B.1.1, and AY.122 lineages of the SARS-CoV-2 virus, respectively.

**Figure 4:**
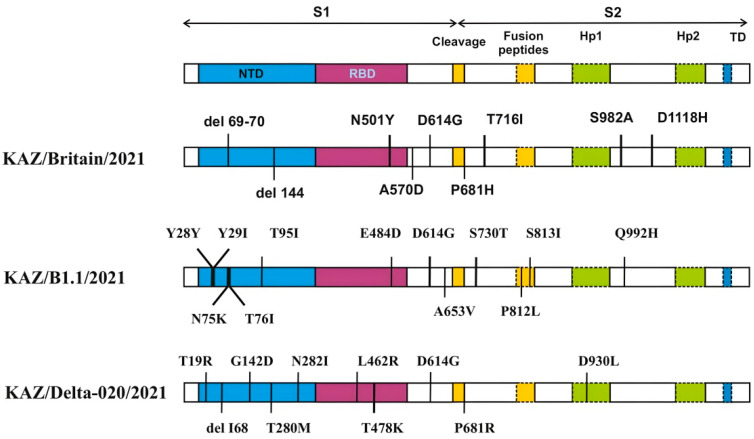
Amino acid changes in the S protein of the studied strains. Description of the SARS-CoV-2 spike mutation in different virus isolates: KAZ/Britain/2021, KAZ/B1.1/2021 and KAZ/Delta020/2021. Colored bars describe the structural domain of the spike protein. NTD—N-terminal domain (blue); RBD—receptor-binding domain (dark purple); Fusion peptides—(orange colour); Hp1—thermal protein 1 (medium spring green); Hp2—thermal protein 2 (medium spring green). TD—transmembrane domain (blue).

The COVID-19 Genome Annotator tool [20] was used to detect mutations in the obtained nucleotide sequences. According to the COVID-19 Genome Annotator data, a total of 127 mutations were detected in the studied strains compared to the reference strain. The most variable regions in the analysis of the genomic distribution of SNP (Single Nucleotide Polymorphism) and amino acid substitutions were the *ORF1ab* protein, which makes up 2/3 of the SARS-CoV-2 virus (Table 1), and the S protein (Table 2 and Figure 4).

The data presented in Table 1 show that the analysis of mutations in the 5′UTR (untranslated region), *ORF1ab* and 3′UTR regions of the studied isolates revealed a total of 61 variations at the nucleotide level. Among them, 18 nucleotide substitutions and 1 deletion were found in the strain KAZ/Britain/2021, 21 nucleotide substitutions and 1 deletion in the strains KAZ/B1.1/2021, and 20 mutations in the strain KAZ/Delta020/2021. A mutation at position 241 in the 5′UTR region of the virus was detected in all three strains studied and resulted in a C to T nucleotide substitution. A C to T nucleotide substitution at positions 3037 and 14,408 in the *ORF1ab* region was detected in all strains studied and resulted in one silent substitution (SNP silent) at position 106 (F106F) and one missense mutation at position 314 (P314L), respectively. A deletion of one amino acid residue S106 (serine → deletion) was observed in two samples studied (KAZ/Britain/2021 and KAZ/B1.1/2021) in the *NSP6* region.

Table 2 and Figure 4 show the distribution of SNP and amino acid substitutions identified in the S protein of the studied strains. A total of 31 mutations were found in the S protein of the studied strains. Among them, 7 amino acid substitutions and 2 deletions were detected in the KAZ/Britain/2021 strain, 12 amino acid substitutions in the KAZ/B1.1/2021 strain, and 9 amino acid substitutions and 1 deletion in the KAZ/Delta020/2021 strain.

Mutational changes in the virus occurred more often in the *S1* region than in the *S2* region. In the *S1* region of the KAZ/Britain/2021 strain, 2 deletions in the NTD region and 4 amino acid changes were detected, of which 1 mutation belongs to the *RBD* region. In the *S2* region, 3 amino acid changes were detected compared to the reference strain. In the strain KAZ/B1.1/2021, 12 mutations were detected compared to the original strain, such as Y28Y, T29I, N74K, T76I, T95I, E484D, D614G, A653V, S730T, P812L, S813I, and Q992H. In the S protein of the strain KAZ/Delta020/2021, sets of mutations (L452R, T478K, and P681R) were found, which are unique only to the Delta variant.

The distribution of SNPs and amino acid substitutions, in addition to the *ORF1ab* and S proteins, was observed in the *ORF8*, *N*, *ORF7a*, *ORF3a*, *M*, *ORF6*, and *ORF7b* proteins of the studied strains and amounted to 9, 9, 6, 4, 3, 2, and 1 amino acid substitution, respectively (Table 3).

As shown in Table 3, a total of 34 variations were detected in the three isolates compared to the original strain. In the current study, four mutations were detected in the *ORF3a* protein across different samples: one mutation (W149L) in the KAZ/Britain/2021 sample, two mutations (A99V, P240S) in the KAZ/B1.1/2021 strain, and one mutation (S26L) in the KAZ/Delta020/2021 strain. Three mutations were detected in the *M* protein across the strains: two mutations (H125Y and K162N) in the KAZ/B1.1/2021 strain and one mutation (I82T) in the KAZ/Delta020/2021 strain. Two mutations (W27 and NL28KF) were detected in the *ORF6* protein, which were found only in the KAZ/Britain/2021 strain. Six mutations were detected in the *ORF7a* protein: three mutations (A79A, E92K and L116F) were detected in the KAZ/B1.1/2021 strain, and three mutations (P45L, V82A and T120I) were detected in the KAZ/Delta020/2021 strain. Only one mutation (T40I) was detected in the *ORF7b* protein in the KAZ/Delta020/2021 strains. Eight mutations were detected in the *ORF8* protein: four mutations (Q27, R52I, K68, and Y73C) were found in the KAZ/B1.1/2021 strain, and four mutations (F120L, F120L, I212N, and 122) were found in the KAZ/Delta020/2021 strain. Nine mutations were detected in the *N* protein compared to the original virus strain: three mutations in the KAZ/Britain/2021 strain (D3L, RG203KR and S235F), two mutations in the KAZ/B.1.1/2021 strain (RG203K and K388I), and four mutations in the KAZ/Delta020/2021 strain (D63G, R203M, G215C, G312G, and D377Y).

### 3.3. Impact of Mutations on Biological Function of Proteins in the Studied SARS-CoV-2 Samples

The PROVEAN web server was used to assess whether the selected mutations could lead to a potential loss of function or remain neutral. Loss of function occurs when a mutation leads to the formation of a non-functional protein. At the same time, a neutral result means that the protein function is preserved despite the presence of a mutation. The PROVEAN platform is focused only on the analysis of the individual effects of each of the mutations identified in the studied virus isolates (Table 4) [28].

Table 4 shows that the proportion of loss-of-function mutations detected in the three studied genomes (KAZ/Britain/2021, KAZ/B1.1/2021, and KAZ/Delta020/2021) of SARS-CoV-2 was studied, and 5 (P218L, T716I, W149L, R52I, and Y73C), 2 (S813I, and Q992H), and 8 (P77L, L452R, I82T, P45L, V82A, F120L, F120L, and R203M) loss-of-function mutations were identified, respectively. Among the genes in the studied samples, the proportion of loss-of-function mutations was higher in the *S* and *ORF8* genes than in other genes.

### 3.4. Phylogenetic Analysis

**Figure 5:**
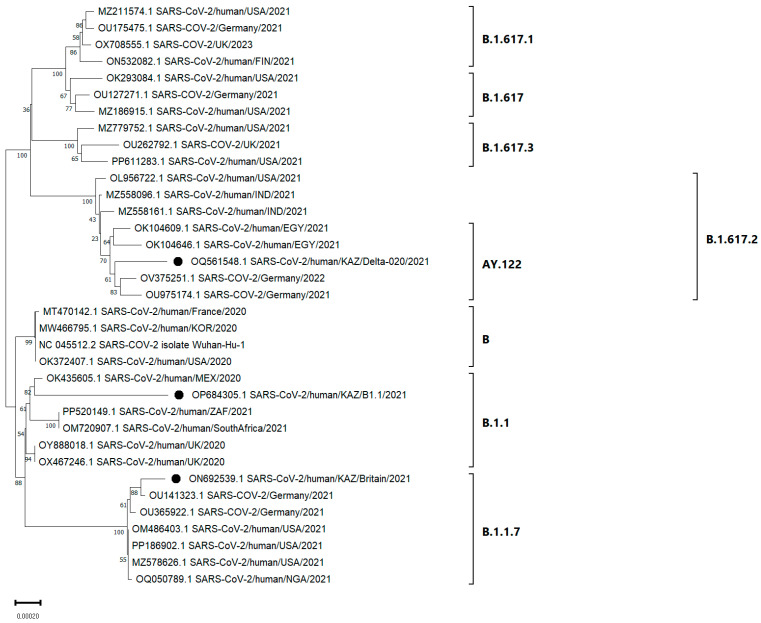
Phylogenetic analysis of the studied strains (black circle) of SARS-CoV-2 and 35 global strains belonging to different virus lineages, such as B.1.617.1, B.1.617.2, B.1.617, B.1.617.3, AY.122, B.1.1.7, B.1.1, and B, which were obtained from the NCBI GenBank database. Here, the x-axis represents the scale of the tree. The studied SARS-CoV-2 virus samples belonging to different lineages are indicated by circles.

Phylogenetic analysis between the studied isolates and other isolates belonging to different lineages of the SARS-CoV-2 virus from the international GenBank NCBI database are presented in Figure 5.

Based on the phylogenetic analysis, the studied strains KAZ/Britain/2021, KAZ/B1.1/2021 and KAZ/Delta020/2021 belong to different SARS-CoV-2 lineages. KAZ/Britain/2021 formed a group (bootstrap (BS) = 100%) with isolates belonging to the B.1.1.7 SARS-CoV-2 lineage. The nucleotide identity between them ranged from 99.96 to 99.97 percent. Within the monophylogenetic group, OU141323.1 SARS-CoV-2/Germany/2021 was the most similar to KAZ/Britain/2021, with a nucleotide similarity of 99.97%. KAZ/B1.1/2021 groups (bootstrap (BS) = 100%) with various samples belonging to the B.1.1 SARS-CoV-2 lineage. KAZ/B1.1/2021 closely matched the samples from Mexico (OK435605.1), showing a nucleotide identity of 99.84%. KAZ/Delta020/2021 formed a monophyletic group (bootstrap (BS) = 61% and (BS) = 100%) with samples that belong to the AY.122 and B.1.617.2 lineages, respectively. However, our KAZ/Delta020/2021 showed high similarity to isolates from Germany (OV375251.1 and OU975174.1), which had a nucleotide identity of 99.94%.

## 4. Discussion

Cleaveland S. et al. reported that most viruses infecting humans are zoonotic. Zoonotic viruses, after entering a cell, adapt inefficiently to a new host and replicate and transmit slowly [29]. Their transmission from animal to human and from human to human depends on many factors, including potential adaptive evolution to virulent strains [30].

RNA viruses are characterized by higher replication fidelity (∼10^−4^ error/site/cycle) and genetically diverse RNA polymerases [31]. However, when RNA viruses circulate in the community, genetic changes continuously occur due to copying errors of RNA polymerase. This, in turn, leads to mutations in the genome [32]. Lee et al. analyzed the rate of genome evolution of several SARS-CoV-2 virus strains over one month and found that the average evolution rate ranged from 1.7926 × 10^−3^ to 1.8266 × 10^−3^ substitutions per site per year [33,34], but four months after the pandemic, the mutation rate of the whole SARS-CoV-2 virus genome was 3.95 × 10^−4^ per nucleotide per year [35].

The rapid evolution of the SARS-CoV-2 genome highlights the need to develop antiviral drugs against the virus [36]. To develop effective antiviral drugs, it is necessary to determine which variant is most actively circulating in society during the pandemic. This depends on the data collected on COVID-19 infection, the epidemiological features among different population groups, as well as the patterns of viral spread in different areas. The modern approach to the use of genomic and information technologies in epidemiological surveillance of SARS-CoV-2 pathogens occupies an important place in measures to prevent and control the virus [37].

Sanger sequencing is considered the most optimal method for sequencing short fragments (<1000 bp) and is useful for filling gaps in partial whole genomes [38,39]. An important step for the successful implementation of the Sanger method is the production of a PCR amplicon from the samples under study and the development of sequencing oligonucleotide primers for the amplification of this PCR amplicon [32].

It is impossible to obtain the complete genomic nucleotide sequence of SARS-CoV-2 virus in a single reaction using the Sanger method. Therefore, in our current study, we designed a set of sequencing primers targeting SARS-CoV-2 virus to obtain the complete genomic nucleotide sequence [18]. The specific designed sequencing primers were selected based on the Wuhan-Hu-1 reference strain, and each designed primer pair overlapped with each other and their sequence was conserved among all SARS-CoV-2 virus variants. The length of the sequencing primers ranged from 600 bp to 772 bp with a GC content of 38% to 50%, and the melting temperature was in the range of 55–57 °C. The PCR products of the studied samples were obtained using the designed sequencing primers.

The length of the amplicons obtained (Figure 1, Figure 2 and Figure 3) corresponds to the length of the synthesized sequencing primers. The developed specific primers covered 100% and amplified the entire genome of the studied samples. After sequencing, the nucleotide sequences of the studied samples were obtained and analyzed in the Pangolin COVID-19 database [19]. According to the Pangolin COVID-19 data, the KAZ/Britain/2021 strain belongs to the B.1.1.7 lineage (Alpha variant). Alpha differs from other variants of the virus by the presence of mutations in the S protein, such as deletion 69–70, deletion 144, N501Y, A570D, D614G, P681H, T716I, S982A, and D1118H [40,41]. The mutations identified in the *S* gene of the studied isolate KAZ/Britain/2021 are 100% consistent with the mutations found in the *S* gene of the alpha variant (Table 2 and Figure 4).

Bo Meng et al. suggest that the H69-V70 deletion in the NTD region of the *S1* subunit of the spike protein, found in the studied SARS-CoV-2 virus sample, is associated with increased infectivity and evasion of the host immune response [42,43,44]. Weng S. et al. confirmed that the Δ144/145 deletion blocks the binding sites of neutralizing antibodies, which is important in preventing the virus from entering the cell and possibly interfering with its replication [44,45]. Some studies describe H69-V70, N501Y, and P681H, which may affect viral infectivity [42,46]. N501Y increases viral infectivity by 70–80% and enhances the binding affinity of the viral S protein to human ACE2 [42,46,47,48]. According to some studies, mutations A570D, T716I, S982A, and D1118H are the result of accumulated mutations of the virus in the community environment, which together increase the lethality and transmissibility of the SARS-CoV-2 virus [48,49]. D614G was found in all three isolates and has become the most common mutation among SARS-CoV-2 variants during the global pandemic [50]. Lubinski B. et al. showed that P681H can increase its cleavage by furin-like proteases, although this process does not lead to viral entry [51]. According to Pangolin COVID-19 data, strain KAZ/B1.1/2021 belongs to the B.1.1 lineage. Currently, the SARS-CoV-2 virus is divided into two lineages: A and B. Lineage B includes 47 different lineages, and Lineage B.1.1 is part of this lineage [52,53,54]. 

The strain KAZ/Delta020/2021, according to Pangolin COVID-19, belongs to the AY.122 lineage (Delta variant, B.1.617). As indicated by the source SARS-CoV-2 Lineage Tree, B.1.617 is divided into three sublineages: B.1.617.1, B.1.617.2, and B.1.617.3. Dhawan M. et al. note that the B.1.617.2 lineage emerged during the second wave of coronavirus infection in India. The B.1.617.2 lineage includes 134 different lineages, one of which is AY.122. Some literature sources emphasize that B.1.617.2 is characterized by differences from other viral variants due to a unique set of mutations, such as L452R, T478K, and P681R. These mutations make it particularly infectious and resistant to neutralizing antibodies in previously infected or vaccinated individuals [55,56,57,58]. Other studies have also shown that the T19R, T478K, P681R, and D950N mutations found in the S gene enhance viral replication and help it evade the host’s immune response [59,60].

The resulting nucleotide sequences were tested using the COVID-19 Genome Annotator [20] to detect mutations. According to the COVID-19 Genome Annotator, a total of 127 mutations were detected in the isolates tested compared to the reference strain. In these studied isolates, the following types of mutations were encountered: SNP, silent mutations, stop codon, deletion and mutations occurring in the 5′ and 3′ untranslated region in the genome compared to the original strain, and their quantities in the studied genomes were 92, 17, 3, 5, 5, and 5, respectively (Table 1, Table 2 and Table 3 and Figure 4). Analysis of the distribution of SNP in the studied genomes showed that the most common in the *ORF1ab* gene (*n* = 36) and S (*n* = 27). The main silent mutations were found in the *ORF1ab* gene (*n* = 13), in the remaining genes *S*, *ORF7b*, *ORF8* and *N* only one was detected, respectively. Deletions were found only in the S gene (*n* = 3) and ORF1ab (*n* = 2).

The study identified mutations in the 5′UTR (C241T), *ORF1ab* (F106F and P314L) and *S* (D614G) regions that were common to all three isolates studied. The study by Periwal N et al. showed that the C nucleotide at position 241 in the 5′UTR region was replaced by a T nucleotide as early as the summer of 2020 [61]. Kim et al. reported that this mutation in the 5′UTR region may affect the rate of transcription and replication of the SARS-CoV-2 virus [12,62,63]. Some studies predicted that the synonymous F106F mutation identified in the NSP3 region of the *ORF1ab* gene may play a role in mRNA processing, altering the properties of the viral protein [12,63]. The missense mutation P314L, found in the *NSP12* region of the *ORF1b* gene, is considered to be part of the core replication/transcription complex and is a conserved protein in coronaviruses [6,64]. Thus, the P314L mutation affects SARS-CoV-2 RNA replication by participating in the activity of *RdRp (RNA-dependent RNA polymerase)* [65,66]. In addition, *RdRp* plays an important role in the process of SARS-CoV-2 viral replication and transcription [67]. D614G was detected in all three isolates and has become the most common mutation among SARS-CoV-2 variants during the global pandemic [50].

It is important to evaluate the change in function of the mutations identified in the study, which may have effects on viral circulation. Aside from this, further studies on these mutations can contribute to the development of various antiviral drugs against SARS-CoV-2. This study revealed significant changes in amino acids in structural and accessory proteins (P218L and P77L in *ORF1ab*; T716I, S813I, Q992H, and N282I in *S*; W149L in *ORF3a*; I82T in *M*; P45L and V82A in *ORF7a*; R52I, Y73C, and F120L in *ORF8;* R203M in *N*), which may cause functional alterations and affect functional characteristics of the virus.

Phylogenetic analysis of SARS-CoV-2 virus isolates showed that the studied samples belong to different virus lineages. In the study, the KAZ/Britain/2021 strain showed significant similarity to the OU141323.1 SARS-CoV-2/Germany/2021 isolate and formed a group with strains that belong to the B.1.1.7 lineage. The nucleotide similarity between these isolates was 99.97%, indicating their very close genetic relationship. KAZ/B1.1/2021 grouped with various isolates that belong to the B.1.1 lineage. In addition, it showed close similarity to samples obtained from Mexico (OK435605.1) SARS-CoV-2/human/MEX/CMX-51/2020), demonstrating a nucleotide identity of 99.84%. KAZ/Delta020/2021 clustered with isolates belonging to the AY.122 and B.1.617.2 lineages. However, KAZ/Delta020/2021 showed high similarity to isolates from Germany—OV375251.1 and OU975174.1—with 99.94% nucleotide identity. According to Pangolin COVID-19 data, the AY.122 lineage is one of the sublineages of B.1.617. B.1.617 emerged in late 2020 in Maharashtra, India [68]. In mid-June 2021, a mutated Delta variant (B.1.617.2), known as the Delta plus, was identified in India [69].

Therefore, the SARS-CoV-2 samples were fully amplified and sequenced using the developed primers, which allowed us to identify mutations compared to the reference strain Wuhan-Hu-1 (NC_045512.2). Sanger-based whole-genome sequencing of the studied SARS-CoV-2 isolates was successfully demonstrated. The data obtained using molecular genetic methods during the pandemic are of great importance for understanding the biology of the virus, developing new diagnostic and therapeutic methods, and making informed public health decisions. Continued research in this area will allow us to be better prepared for future pandemics.

## Figures and Tables

**Table 1:** Mutations in the 5′UTR, *ORF1ab*, and 3′UTR regions of the studied SARS-CoV-2 virus strains compared to the Wuhan-Hu-1 reference sequence (NC_045512) [16].

Protein	Data for Strain:									Amino Acid Change
	Wuhan-Hu-1 ^a^		KAZ/Britain/2021		KAZ/B1.1/2021		KAZ/Delta020/2021		Type of Mutation	
	Position	Variant	Variant	Position	Variant	Position	Variant	Position		
5′ UTR ^b^	106	C	–	–	T	29	–	–	–	106
	210	G	–	–	–	–	T	184	–	210
	241	C	T	215	T	164	T	215	–	241
*ORF1ab*	344	C	-	-	T	267	–	–	SNP	L27F
	913	C	T	887	–	–	–	–	SNP_silent	S36S
	1048	G	–	–	–	–	T	1022	SNP	K81N ^c^
	1688	A	C	1662	–	–	–	–	SNP	I295L
	1899	G	–	–	–	–	T	1873	SNP	R365L
	2110	C	T	2084	–	–	–	–	SNP_silent	N435N
	2530	A	–	–	G	2453	–	–	SNP_silent	E575E
	3037	C	T	3011	T	2960	T	3011	SNP_silent	F106F
	3267	C	T	3241	–	–	–	–	SNP	T183I
	4181	G	–	–	–	–	T	4155	SNP	A488S
	4449	C	–	–	A	4372	–	–	SNP	T577N
	4455	C	–	–	T	4378	–	–	SNP	A579V
	4475	C	–	–	T	4398	–	–	SNP	R586C
	5388	C	A	5362	–	–	–	–	SNP	A890D
	5829	A	–	–	C	5752	–	–	SNP	K1037T
	5986	C	T	5960	–	–	–	–	SNP_silent	F1089F
	6402	C	–	–	–	–	T	6376	SNP	P1228L
	6954	T	C	6928	–	–	–	–	SNP	I1412T
	7042	G	T	7016	–	–	–	–	SNP	M1441I
	7124	C	–	–	–	–	T	7098	SNP	P1469S
	8986	C	–	–	–	–	T	8960	SNP_silent	D144D
	9053	G	–	–	–	–	T	9027	SNP	V167L
	9749	A	–	–	G	9672	–	–	SNP	K399E
	9867	T	–	–	G	9790	–	–	SNP	L438R
	10,029	C	–	–	–	–	T	10,003	SNP	T492I
	10,198	C	–	–	T	10,121	–	–	SNP_silent	D48D
	11,195	C	T	11,169	–	–	–	–	SNP	L75F
	11,201	A	–	–	–	–	G	11,175	SNP	T77A
	11,288	TCTGGTTTT	del	11,261	del	11,210	–	–	SNP_stop	S106
	11,332	A	–	–			G	11,306	SNP_silent	V120V
	14,120	C	T	14,085			–	–	SNP	P218L
	14,408	C	T	14,373	T	14,322	T	14,382	SNP	P314L
	14,676	C	T	14,641	–	–	–	–	SNP_silent	P403P
	15,017	C	–	–	T	14,931	–	–	SNP	A517V
	15,279	C	T	15,244	–	–	–	–	SNP_silent	H604H
	15,451	G	–	-	–	–	A	15,425	SNP	G662S
	16,176	T	C	16,141	–	–	–	–	SNP	T903T
	16,466	C	–	–	–	–	T	16,440	SNP	P77L
	18,271	G	–	–	–	–	A	18,245	SNP	E78K
	18,337	G	–	–	–	–	T	18,311	SNP	A100S
	19,220	C	–	–	–	–	T	19,194	SNP	A394V
	20,405	C	T	20,370	–	–	–	–	SNP	P262L
	20,759	C	–	–	T	20,673	–	–	SNP	A34V
	21,080	A	–	–	G	20,994	–	–	SNP	K141R
	21,215	A	G	21,180	–	–	–	–	SNP	H186R
	21,446	A	–	–	G	21,360	–	–	SNP	K263R
3′ UTR	27,389	C	–	–	T	27,303	–	–	–	27,389
	29,733	–	–	–	TA	29,648	–	–	–	29,733
	29,742	G	–	–	–	–	T	29,716	–	29,742
	29,755	–	–	–	C	29,672	–	–	–	29,755
	29,790	–	–	–	T	29,708	–	–	–	29,790

^a^ Severe acute respiratory syndrome coronavirus 2 strain Wuhan-Hu-1, complete genome sequence (GenBank accession number NC_045512) [16]. ^b^ UTR, untranslated region. ^c^ K81N, the K-to-N change at Position 81.

**Table 2:** Mutations in the S protein of the studied SARS-CoV-2 virus strains compared to the Wuhan-Hu-1 reference sequence (NC_045512) [16].

Protein	Data for Strain:							
	Wuhan-Hu-1 ^a^		KAZ/Britain/2021		KAZ/B1.1/2021		KAZ/Delta020/2021	
	Position	Variant	Variant	Position	Variant	Position	Variant	Position
*S*	21,618	C	–	–	–	–	G	21592
	21,646	C	–	–	T	21,560	–	–
	21,648	C	–	–	T	21,562	–	–
	21,765	TACATG	del	21,729	–	–	–	–
	21,766	A	–	–	–	–	del	21,739
	21,784	T	–	–	A	21,698	–	–
	21,789	C	–	–	T	21,703	–	–
	21,846	C	–	–	T	21,760	–	–
	21,987	G	–	–	–	–	A	21,961
	21,993	ATT	del	21,951	–	–	–	–
	22,185	C	–	–	–	–	T	22,159
	22,407	A	–	–	–	–	T	22,381
	22,917	T	–	–	–	–	G	22,891
	22,995	C	–	–	–	–	A	22,969
	23,014	A	–	–	C	22,928	–	–
	23,063	A	T	23,019	–	–	–	–
	23,271	C	A	32,227	–	–	–	–
	23,403	A	G	23,359	G	23,317	G	23,377
	23,520	C	-	–	T	23,434	–	–
	23,604	C	A	23,560	–	–	G	23,578
	23,709	C	T	23,665	–	–	–	–
	23,751	C	–	–	T	23,665	–	–
	23,997	C	–	–	T	23,911	–	–
	24,000	G	–	–	T	23,914	–	–
	24,410	G	–	–	–	–	A	24,384
	24,506	T	G	24,462	–	–	–	–
	24,538	A	–	–	T	24,452	–	–
	24,914	G	C	24,870	–	–	–	–

^a^ Severe acute respiratory syndrome coronavirus 2 strain Wuhan-Hu-1, complete genome sequence (GenBank accession number NC_045512).

**Table 3:** Mutations in the remaining proteins of the studied SARS-CoV-2 virus strains compared to the Wuhan-Hu-1 reference sequence (NC_045512) [16].

Protein	Data for Strain:									Amino Acid Change
	Wuhan-Hu-1 ^a^		KAZ/Britain/2021		KAZ/B1.1/2021		KAZ/Delta020/2021		Type of Mutation	
	Position	Variant	Variant	Position	Variant	Position	Variant	Position		
*ORF3a*	25,459	C	–	–	–	–	T	25,443	SNP	S26L ^b^
	25,688	C	–	–	T	25,602	–	–	SNP	A99V
	25,838	G	T	25,794	–	–	–	–	SNP	W149L
	26,110	C	–	–	T	26,024	–	–	SNP	P240S
*M*	26,767	T	–	–	–	–	C	26,741	SNP	I82T
	26,895	C	–	–	T	26,809	–	–	SNP	H125Y
	27,008	G	–	–	T	26,922	–	–	SNP	K162N
*ORF6*	27,281	GG	AA	27,237	–	–	–	–	SNP_stop	W27
	27,285	TC	AT	27,241	–	–	–	–	SNP	NL28KF
*ORF7a*	27,527	C	–	–	–	–	T	27,501	SNP	P45L
	27,638	T	–	–	–	–	C	27,612	SNP	V82A
	27,630	C	–	–	T	27,544	–	–	SNP_silent	A79A
	27,667	G	–	–	A	27,581	–	–	SNP	E92K
	27,739	C	–	–	T	27,653	–	–	SNP	L116F
	27,752	C	–		–	–	T	27,726	SNP	T120I
*ORF7b*	27,874	C	–	–	–	–	T	27,848	SNP	T40I
*ORF8*	27,919	T	–	–	–	–	C	27,893	SNP	I9T
	27,972	C	T	27,928	–	–	–	–	SNP_stop	Q27
	28,048	G	T	28,004	–	–	–	–	SNP	R52I
	28,095	A	T	28,051	–	–	–	–	SNP_stop	K68
	28,111	A	G	28,067	–	–	–	–	SNP	Y73C
	28,251	T	–	–	–	–	C	28,225	SNP	F120L
	28,253	C	–	–	–	–	A	28,227	SNP	F120L
	28,255	T	–	–	–	–	A	28,229	SNP	I121N
	28,258	A	–	–	–	–	G	28,232	SNP_silent	122 *
*N*	28,280	GAT	CTA	28,236	–	–	–	–	SNP	D3L
	28,461	A	–		–	–	G	28,435	SNP	D63G
	28,881	GGG	AAC	28,837	AAC	28,837	–	–	SNP	RG203KR
	28,881	G	–	–	–	–	T	28,855	SNP	R203M
	28,916	G	–	–	–	–	T	28,890	SNP	G215C
	28,977	C	T	28,933	–	–	–	–	SNP	S235F
	29,236	C	–	–	–	–	T	29,210	SNP_silent	G312G
	29,402	G	–	–	–	–	T	29,376	SNP	D377Y
	29,436	A	–	–	T	29,350	–	–	SNP	K388I

^a^ Severe acute respiratory syndrome coronavirus 2 strain Wuhan-Hu-1, complete genome sequence (GenBank accession number NC_045512). ^b^ S26L, the S-to-L change at Position 26. 122 *, the studied strain according to the Pangolin database belongs to the AY.122 lineage.

**Table 4:** Estimates of various mutations in the genomes of the studied SARS-CoV-2 strains.

Protein	KAZ/Britain/2021			KAZ/B1.1/2021			KAZ/Delta020/2021		
	Amino Acid Change	PROVEAN Assessment	The Effect of Variation on Protein	Amino Acid Change	PROVEAN Assessment	The Effect of Variation on Protein	Amino Acid Change	PROVEAN Assessment	The Effect of Variation on Protein
*ORF1ab*	I295L	0.232	Neutral	L27F	−0.047	Neutral	K81N	−0.070	Neutral
	T183I	0.216	Neutral	A579V	0.011	Neutral	R365L	−0.939	Neutral
	T577N	0.240	Neutral	R586C	−0.727	Neutral	A488S	−0.061	Neutral
	A890D	−1.749	Neutral	K1037T	−1.196	Neutral	P1228L	−1.038	Neutral
	I1412T	−0.370	Neutral	K399E	−1.877	Neutral	P1469S	0.338	Neutral
	M1441I	0.263	Neutral	L438R	0.659	Neutral	V167L	−0.696	Neutral
	L75F	−2.290	Neutral	P314L	−0.446	Neutral	T492I	1.435	Neutral
	P218L	−5.021	Deleterious	A517V	−1.291	Neutral	T77A	−0.878	Neutral
	P314L	−0.446	Neutral	A34V	1.158	Neutral	P314L	−0.446	Neutral
	P262L	−0.014	Neutral	K141R	−0.221	Neutral	G662S	−2.475	Neutral
	H186R	−0.267	Neutral	K263R	−1.344	Neutral	P77L	−6.845	Deleterious
	–	–	–				E78K	−1.123	Neutral
	–	–	–	–	–	–	A100S	1.338	Neutral
	–	–	–	–	–	–	A394V	−1.523	Neutral
*S*	H69del	0.260	Neutral	Y28Y	0.000	Neutral	T19R	−0.839	Neutral
	Y145del	0.853	Neutral	T29I	−1.538	Neutral	I68del	−0.821	Neutral
	N501Y	−0.090	Neutral	N74K	−1.309	Neutral	G142D	−0.277	Neutral
	A570D	−0.682	Neutral	T76I	−0.115	Neutral	T208M	−0.314	Neutral
	D614G	0.598	Neutral	T95I	−1.214	Neutral	N282I	−3.717	Deleterious
	P681H	0.060	Neutral	E484D	−0.210	Neutral	L452R	0.559	Neutral
	T716I	−3.293	Deleterious	D614G	0.598	Neutral	T478K	−0.524	Neutral
	S982A	−1.505	Neutral	A653V	−0.715	Neutral	D614G	0.598	Neutral
	D1118H	−1.142	Neutral	S730T	−0.040	Neutral	P681R	0.741	Neutral
	–	–	–	P812L	−0.868	Neutral	D950N	−1.631	Neutral
	–	–	–	S813I	−2.867	Deleterious	–	–	–
	–	–	–	Q992H	−4.059	Deleterious	–	–	–
*ORF3a*	–	–	–	–	–	–	S26L	−2.314	Neutral
				A99V	−1.962	Neutral	–	–	–
	W149L	−9.419	Deleterious		–	–	–	–	–
	–	–	–	P240S	−1.495	Neutra	–	–	–
*M*	–	–	–				I82T	−3.853	Deleterious
	–	–	–	H125Y	0.799	Neutral	–	–	–
	–	–	–	K162N	0.501	Neutral	–	–	–
*ORF7a*	–	–	–	–	–	–	P45L	−10.000	Deleterious
	–	–	–	–	–	–	V82A	−2.667	Deleterious
	–	–	–	A79A	0.000	Neutral	–	–	–
	–	–	–	E92K	−1.842	Neutral	–	–	–
	–	–	–	L116F	−1.263	Neutral	–	–	–
	–	–	–	–	–	–	T120I	−1.789	Neutral
*ORF7b*	–	–	–	–	–	–	T40I	−2.000	Neutral
*ORF8*				–	–	–	I9T	−1.333	Neutral
	R52I	−6.417	Deleterious	–	–	–	–	–	–
	Y73C	−4.500	Deleterious	–	–	–	–	–	–
	–	–	–	–	–	–	F120L	−2.667	Deleterious
	–	–	–	–	–	–	F120L	−2.667	Deleterious
	–	–	–	–	–	–	I121N	−0.667	Neutral
*N*	D3L	−0.230	Neutral	–	–	–			
	–	–	–	–	–	–	D63G	−0.929	Neutral
	–	–	–	–	–	–	R203M	−3.304	Deleterious
	–	–	–	–	–	–	G215C	−0.953	Neutral
	–	–	–	–	–	–	S235F	−1.738	Neutral
	–	–	–	–	–	–	D377Y	−1.779	Neutral
	–	–	–	K388I	−1.204	Neutral	–	–	–

The threshold value was set at −2.5.

## Data Availability

The complete genome sequence of SARS-CoV-2 in this study is available in GenBank under accession numbers ON692539.1, OP684305.1 and OQ561548.1.
